# Rasch validation of the Arabic version of the beach center family quality of life scale (BCFQOL-AR)

**DOI:** 10.3389/fpsyg.2022.984664

**Published:** 2022-11-03

**Authors:** Ghaleb Hamad Alnahdi

**Affiliations:** Special Education Department, Prince Sattam Bin Abdulaziz University, Al-Kharj, Saudi Arabia

**Keywords:** Rasch analysis, family quality of life, Saudi Arabia, unidimensionality, validity, psychometric properties

## Abstract

**Aim:**

This study aimed to examine the dimensionality of the BCFQOL-AR using Rasch analysis.

**Method:**

The sample consisted of 320 families having a member with intellectual or developmental disabilities. Rasch analysis was used to validate the dimensionality of the scale. The participants were from Riyadh, Saudi Arabia.

**Results:**

The BCFQOL-AR 25-item scale was multidimensional. Rasch analyses support the unidimensionality of the five subscales. There were no indicators of differential item function for any of the items, regardless of sex or age.

**Conclusion:**

The BCFQOL-AR is a multidimensional scale that measures families with members who are satisfied with their quality of life. Therefore, obtaining a total score at the subscale level is supported and showed that each of the five subscales of the BCFQOL can be used alone. This study partially supports the practices used with other versions of the scale, by providing the statistical base, where means were used at the subscale level in different countries.

## Introduction

The topic of quality of life has recently attracted the attention of researchers. The same applies to research related to individuals with disabilities and the quality of life of their families (Boelsma et al., 2017; Vanderkerken et al., 2019). From this perspective, the importance of measuring the level of quality of life of families of individuals with disabilities is clear.

Few reliable scales can be used for disability-related studies. This is especially true for new topics, such as quality of life and the Arabic language. The BCFQOL scale is one of the most used scales with different samples from different cultures (Park et al., 2003; Poston et al., 2003; Verdugo et al., 2005; Zuna et al., 2009; Balcells-Balcells et al., 2011; Meral et al., 2013; Parpa et al., 2016; Chiu et al., 2017; Schlebusch et al., 2017; Waschl et al., 2019; Balcells-Balcells et al., 2020; Kyzar et al., 2020). Psychometric properties were examined and showed good reliability and construct validity (Hoffman et al., 2006), for example, in China, Hu et al. (2012) findings supported the five-dimensional factor structure, and Chiu et al., 2017 results showed good fit indices for the five-factor model (first-order) with correlations between the five latent variables ranging from 0.70 to 0.93. In addition, similar findings with good psychometric properties in other different studies, such as, in the US (Boehm et al., 2015), Turkey (Meral et al., 2013), and Saudi Arabia (Alnahdi et al., 2021). In this context, it is important to note that there are few studies that have reached different conclusions regarding the number of factors of the scale (Zuna et al., 2009; Balcells-Balcells et al., 2020; Kyzar et al., 2020). For example, Kyzar et al. (2020) found that 3 factors were supported (from 4 factor because the fifth subscale was removed and 21 items only used). However, all these studies examined psychometric properties using only the classical test theory and the dimensionally of the scale and the subscale were not examined. It is important to check the scale’s unidimensionality, as “the use of the total score as a summary of a person’s value on the variable implies that persons can be compared by their total scores, or the estimates of their true scores, and this implies a unidimensional construct” (Andrich and Marais, 2019, p. 50). In this study, we examined psychometric properties using Rasch analysis based on the item response theory. Rasch analysis allows for examination through a unified approach (Tennant and Conaghan, 2007). For instance, it allows us to examine the unidimensionality of the scale, hierarchy of items from difficult to easy to endorse, the suitability of category structure, and finding misfitting persons and items. In addition, it offers the benefit of a person-item map for easy visual comparison of item difficulty and participants’ ability (Lee et al., 2010; Cappelleri et al., 2014). In addition, “the Wright map of item–person relationship, available only for the family of Rasch models, has the distinct benefit of aligning person measures as well as item measures on the same logit scale” (Cheng et al., 2009, p. 378). In sum, “Rasch analysis is a powerful tool for evaluating construct validity” (Baghaei, 2008, p. 1146).

Researchers used the total score as an indicator of the FQOL satisfaction level based on the overall mean score for all 25 items (e.g., Boehm et al., 2015; Chiu et al., 2017). According to Boehm et al. (2015), the use of the overall mean to examine predictors was justified because subscales were highly correlated. In addition, some studies reported the overall Cronbach’s alpha (Hoffman et al., 2006). However, there is no statistical evidence from the previous studies to support the overall mean. Hence, the importance of this study is to examine whether there is statistical evidence that supports the use of an overall mean, or the use of averages at the level of subscales.

In summary, this study examined two aspects. First, whether “each of the five subscales was shown to be unidimensional and internally consistent” (Hoffman et al., 2006, p. 1080) for the Arabic version (Alnahdi et al., 2021) using Rasch analysis. Second, whether having total scores on the BCFQOL scale would be supported using Rasch analysis, which would allow us to examine psychometric properties that are not visible in the classical test theory (Tennant and Conaghan, 2007; Lee et al., 2010; De Ayala, 2013; Cappelleri et al., 2014; Bond and Fox, 2015).

## Materials and methods

### Sample and instrument

This study sample comprised 320 families who had a member with developmental disabilities. Data were collected from the Riyadh region of Saudi Arabia. Participation in the study was voluntary. Based on the type of disability, approximately 48% of the families had a member with intellectual disability, 19% had a member with autism, 9.7% had a member with a physical disability, and the rest had other disabilities. Approximately, 26% of the participants were fathers and 27% were mothers, about 27% were brothers or sisters, and the rest were other relatives of the individual with disability. The participants’ age ranged from 18 to 70 years (*M* = 34, *SD* = 13). The translation and validation *via* confirmatory factor analysis were confirmed for the Arabic version of the BCFQOL (Alnahdi et al., 2021). After obtaining approval from the IRB committee at the university. The staff of the schools where students with disabilities are taught have been contacted. Coordination was made with the school to deliver the paper questionnaire by the school and collect it from parents. This scale contains 25 items within five subscales: Family Interaction (e.g., “My family members talk openly with each other”), Parenting (e.g., “Family members teach the children how to get along with others”), Emotional Well-being (e.g., “My family members have friends or others who provide support”), Physical/Material Well-being (e.g., “My family gets medical care when needed”), and Disability-Related Support (“My family member with special needs has support to make progress at school or workplace”). Responses were rated on a five-point Likert scale with the options “very dissatisfied,” “dissatisfied,” “neither,” “satisfied,” and “very satisfied.” The fit indices supported the hypothesized five-factor model (Alnahdi et al., 2021). Additionally, the five subscales showed good reliability (α = 0.854 to 0.946) and McDonald’s Omega ranged from 0.832 to 0.958.

### Rasch analysis

Rasch analysis in this study followed the guidelines recommended by Tennant and Conaghan (2007). The Rasch Unidimensional Measurement Model (RUMM2030) software (Andrich et al., 2009) was used for all analyses in this study. A non-significant Chi-square and mean close to zero for items and person residuals were considered good indicators of the overall fit. Threshold maps and response category curves were checked for items with threshold disorder. Such items are used to solve this issue (Tennant et al., 2004; Tennant and Conaghan, 2007). “For a well-fitting item, one would expect that, across the entire range of the traits being measured, each response option would systematically take turns showing the highest probability of endorsement” (Pallant and Tennant, 2007, p. 6). The item and person statistics were checked for residuals outside the range ±2.5, and a significant Chi-square value (using Bonferroni corrected *p*-values) was considered to be a misfit. Correlations of items’ residuals were checked for local dependency indicators by looking for correlations that were above the average of other items’ residuals correlations by 0.20, as an indicator of local dependency (Hissbach et al., 2011; Makransky and Bilenberg, 2014; Christensen et al., 2017; Lundgren-Nilsson et al., 2019).

Internal consistency was checked *via* the person separation index (PSI) and determined at a value of 0.7 or higher (Tennant and Conaghan, 2007). The differential item function (DIF) was checked to ensure that items performed similarly regardless of participants’ gender or age (Pallant and Tennant, 2007). The unidimensionality of the scale was examined using Smith’s test (Smith, 2002). Two ability estimates were calibrated for each person, and only if there were significant differences between these two estimates in 5% of the sample or less, or the lower limit of the binomial 95% confidence interval of proportions was at the 5% level or less, it was considered an indicator of a unidimensional scale (Smith, 2002; Tennant and Conaghan, 2007; Alnahdi, 2018). The two estimates were calibrated by dividing the items into two sets. Based on these two sets, we obtained two estimates for each participant. The first set of items included items that loaded positively in the first factor on the principal component analysis of the residuals, and the second set included items that loaded negatively on the first principal component analysis of the residuals. In the final step, we transferred raw scores to the interval scores. The formula used was as follows: “*Y* = *M* + (S × logit score). *S* = range of interval-level scale [(60; for a 0 to 60 scale)] divided by the actual range of logit scores, and *M* = (minimum score of interval-level scale) – (minimum logit score × S)” (Alnahdi, 2018, p. 355). This helped in interpreting differences in scores, as the interval score is easier to interpret with equal weight for each unit across the scale (Alnahdi, 2018, 2019).

## Results

The default model in RUMM2030, the partial credit model, was used to analyze the study data. Thus, thresholds were calibrated for each item. First, we conducted a Rasch analysis of all items (25) to examine the model fit. The Chi-square test was significant, and the dimensional test was far from the recommended 5% (24%) (Table 1).

**Table 1:** Rasch analysis statistics at each step.

			Item residual fit		Person residual fit		Item-trait interaction			Unidimensionality *t*-tests	
	# Of items	*N*	Mean	*SD*	Mean	*SD*	χ^2^ (*df*)	*p*	PSI	% Significant tests	Lower limit of 95% *CI*
Initial analysis	25	320	−0.25	2.68	−0.57	2.08	317.3 (100)	0.000	0.941	24.1%	19.5%
Rescoring 22 items	25	320	0.03	2.59	−0.46	2.08	353.7 (100)	0.000	0.943	22.19%	17.8%
1^st^ subscale	6	320	−0.21	1.03	−0.72	1.71	26.86 (24)	0.310	0.875	**2.81%**	**1.3%**
1^st^ subscale (item 4 rescored)	6	320	−0.05	1.10	−0.66	1.62	26.44 (24)	0.330	0.879	**3.75%**	**2%**
2^nd^ subscale	6	320	−0.52	1.06	−0.64	1.57	24.36 (24)	0.441	0.848	**2.50%**	**1%**
2^nd^ subscale (rescored 6 items)	6	320	−0.17	1.28	−0.79	2.04	28.44 (24)	0.241	0.851	**4.69%**	**2.6%**
3^rd^ subscale	4	320	−0.05	1.53	−0.69	1.59	25.85 (16)	**0.056**	0.769	**2.50%**	**1%**
3^rd^ subscale (rescored item 13)	4	320	0.03	1.53	−0.64	1.45	24.95 (16)	**0.070**	0.770	**3.13%**	**1.5%**
4^th^ subscale	5	320	−0.35	1.58	−0.74	1.71	26.08 (20)	**0.163**	0.749	**1.88%**	**0.7%**
4^th^ subscale (rescored all 5 items)	5	320	−0.07	1.69	−0.90	1.99	22.78 (20)	**0.299**	0.751	**1.88%**	**0.7%**
5^th^ subscale	4	320	−0.16	1.85	−0.83	1.78	25.22 (16)	**0.066**	0.826	**2.19%**	**0.9%**
Five subscales	5	320	0.30	2.09	−0.42	1.07	38.48 (20)	0.00*	0.866	**4%**	**2.1%**
Ideal values			**0.0**	**<1.4**	**0.0**	**<1.4**		**>0.05**	**>0.7**	**≤5%**	**≤5%**

1^st^ subscale, family interaction; 2^nd^ subscale, parenting; 3^rd^ subscale, emotional well-being; 4^th^ subscale, physical/material well-being; 5^th^ subscale, disability-related support; *, by adjusting the sample to 200, the *p* > 0.05, bold, indicator for unidimensionality.

**Figure 1:**
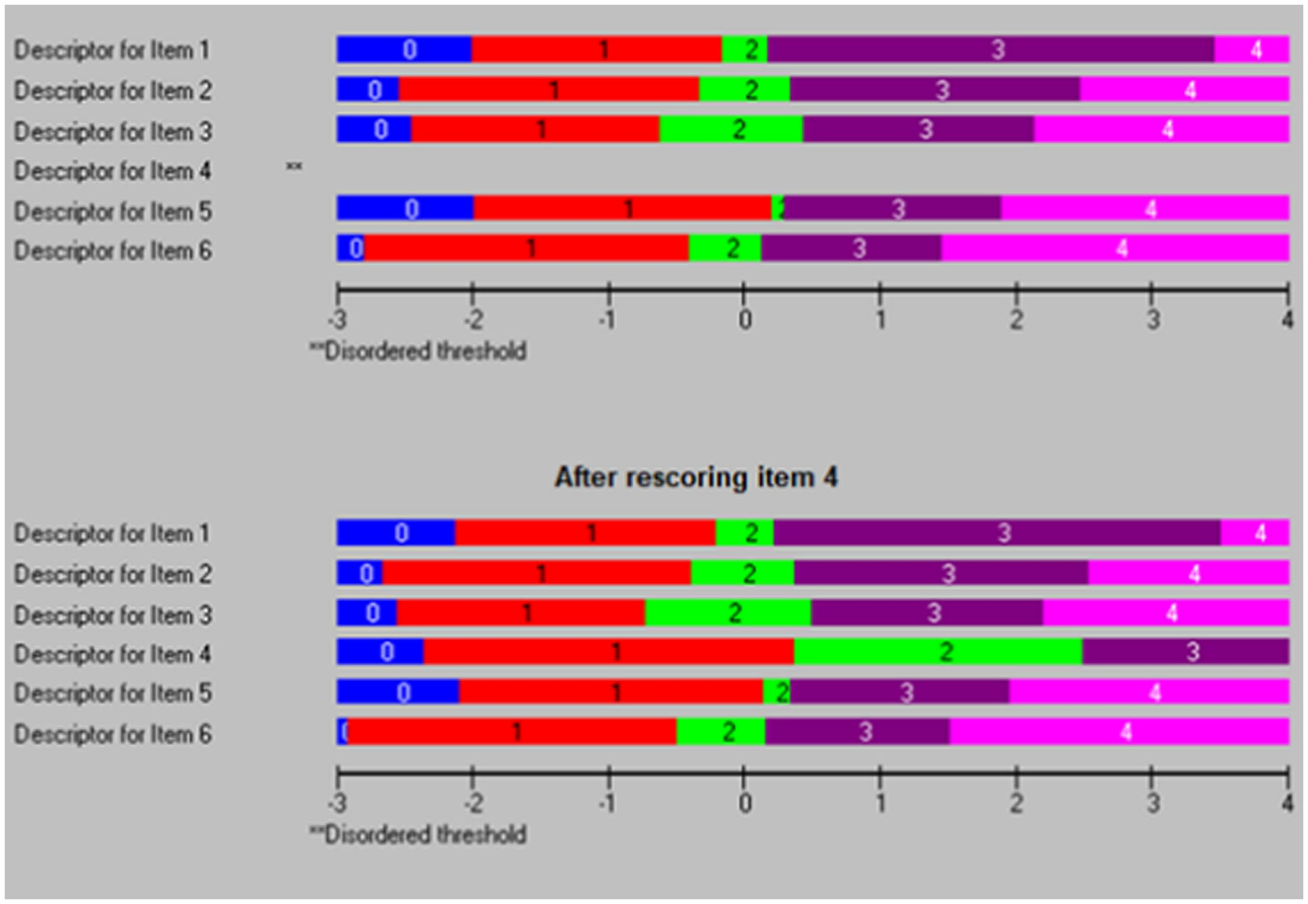
Thresholds map for family interaction before (top chart) and after rescoring of item 4 (bottom chart).

**Figure 2:**
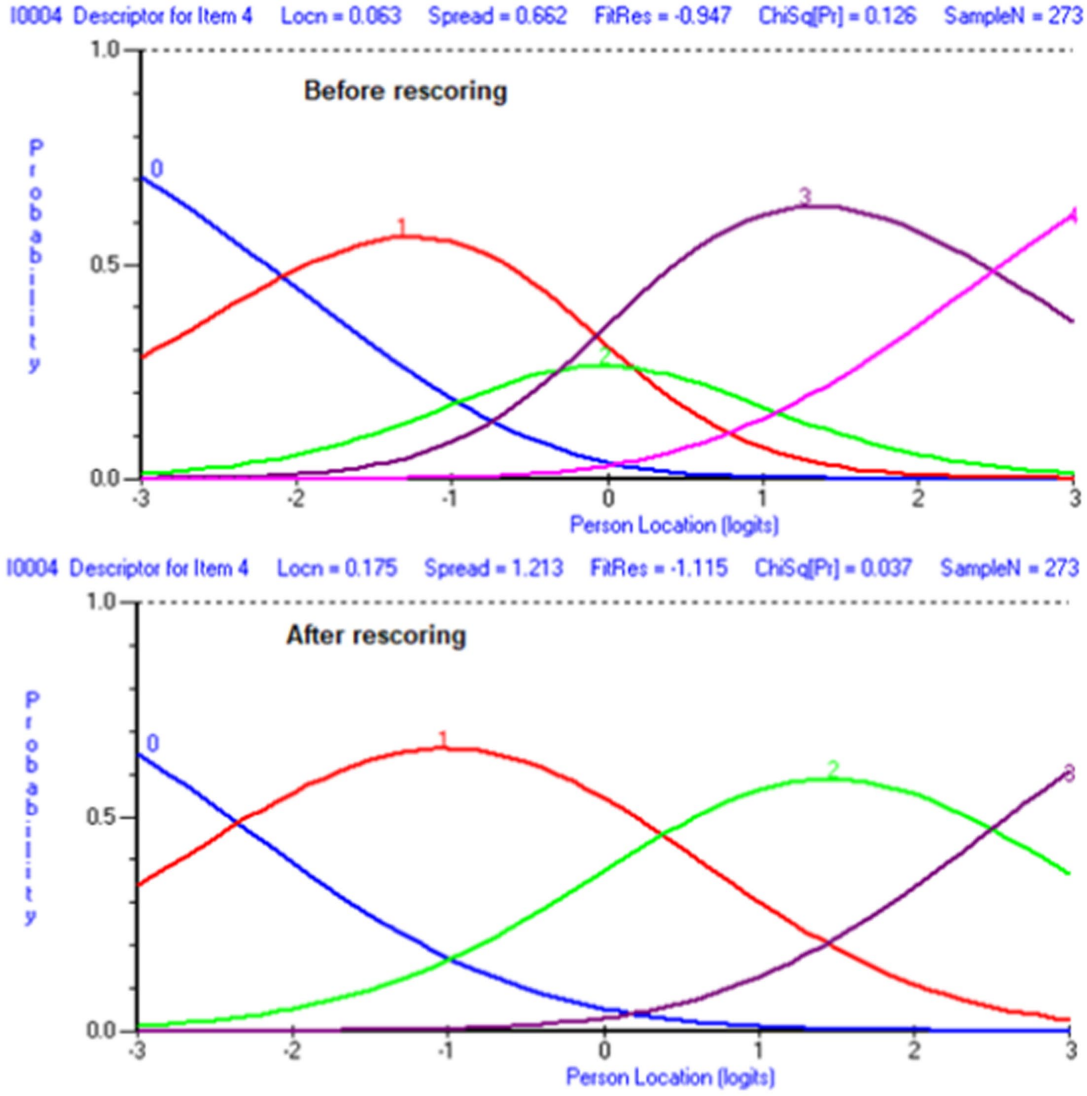
Item 4 ICC before and after rescoring.

Next, we rescored 13 items that showed threshold disorders (Figure 1). However, this did not improve the Chi-square statistics and did not solve the dimensionality issue with 22% significant t-tests in the dimensionality test. Subsequently, we conducted a separate Rasch analysis for each subscale. For the first subscale (family interaction), good indicators were shown with a non-significant Chi-square and supported the dimensionality, with only 2.8% of *t*-tests being significant in the dimensionality test. After that, the threshold map for this scale was checked, and we found that item 4 showed threshold disorder (Figure 2). We re-scored item 4 and re-ran the Rasch analysis. In addition, all the indicators supported the unidimensionality of the first subscale. Similar steps were followed for the other four subscales. All items were re-scored in the second subscale (parenting), one item (13) was re-scored in the third subscale (emotional well-being), all items were rescored for the fourth subscale (physical well-being), and no items were recorded for the fifth subscale (disability-related support). All subscales showed a very good level of internal consistency, with PSI scores ranging from 0.770 to 0.879. Table 2 shows the final scale with the new scoring in the final validated scale. All five subscales met the unidimensionality criteria, with a significance of less than 5% in t-tests. Lastly, we clustered all items within each subscale in one testlet “super item” to represent each domain, conducted the Rasch analysis on five subscales, and examined the unidimensionality of this model. The unidimensionality test supported the fit of this model, with a significance of less than 5% in the *t*-test.

**Table 2:** The final Rasch-validated scale.

Item			Strongly dissatisfied	Dissatisfied	Not sure	Satisfied	Strongly satisfied
1	My family enjoys spending time together	Family Interaction	0	1	2	3	4
2	My family members talk openly with each other		0	1	2	3	4
3	My family solves problems together		0	1	2	3	4
4	**My family members support each other to accomplish goals**		**0**	**1**	**1**	**2**	**3**
5	My family members show that they love and care for each other		0	1	2	3	4
6	My family is able to handle life’s ups and downs		0	1	2	3	4
7	**Family members help the children learn to be independent**	Parenting	**0**	**1**	**1**	**2**	**3**
8	**Family members help the children with schoolwork and activities**		**0**	**1**	**1**	**2**	**3**
9	**Family members teach the children how to get along with others**		**0**	**1**	**1**	**2**	**3**
10	**Adults in the family teach the children to make good decisions**		**0**	**1**	**1**	**2**	**3**
11	**Adults in the family know other people in the children’s lives (i.e., friends, teachers)**		**0**	**1**	**1**	**2**	**3**
12	**Adults in the family have time to take care of the individual needs of every child**		**0**	**1**	**1**	**2**	**3**
13	**My family gets the support they need to relieve stress**	Emotional well-being	**0**	**1**	**1**	**2**	**3**
14	My family members have friends or others who provide support		0	1	2	3	4
15	My family members have some time to pursue their own interests		0	1	2	3	4
16	My family has outside help available to take care of special needs of all family members		0	1	2	3	4
17	**My family members have transportation to get to the places they need to be**	Physical/material well-being	**0**	**1**	**1**	**2**	**3**
18	**My family gets dental care when needed**		**0**	**1**	**1**	**2**	**3**
19	**My family gets medical care when needed**		**0**	**1**	**1**	**2**	**3**
20	**My family has a way to take care of our expenses**		**0**	**1**	**1**	**2**	**3**
21	**My family feels safe at home, work, school, and in our neighborhood**		**0**	**1**	**1**	**2**	**3**
22	My family member with special needs has support to make progress at school or workplace	Disability-related support	0	1	2	3	4
23	My family member with special needs has support to make progress at home		0	1	2	3	4
24	My family member with special needs has support to make friends		0	1	2	3	4
25	My family has a good relationship with the service providers who work with our family member with a disability		0	1	2	3	4

Boldface = rescored items.

The item statistics for all items within each subscale are shown in Table 3. For example, in the family interaction subscale item 1, “*My family enjoys spending time together,*” was the most difficult item to endorse while item 6, “*My family is able to handle life’s ups and downs,*” was the easiest. In the parenting subscale item 9, “*Family members teach the children how to get along with others,*” was the most difficult to endorse, while item 11, “*Adults in my family know other people in the children’s lives* (i.e.*, friends, teachers*),” was the easiest to endorse. In the emotional well-being subscale, item 13, “*My family has the support we need to relieve stress,*” was the most difficult to endorse. Item 14, *“My family members have friends or others who provide support,”* was the easiest. In the physical/material subscale, item 19, “*My family gets medical care when needed,*” was the most difficult to endorse, while item 21, “*My family feels safe at home, work, school, and in our neighborhood,*” was the easiest. In the disability-related support subscale, item 22, “*My family member with special needs has support to make progress at school or workplace,*” was the most difficult to endorse, while item 25, “*My family has a good relationship with the service providers who work with our family member with a disability,*” was the easiest.

**Table 3:** Item-fit statistics in the Arabic version of FQOL scale sorted from descending based on items location (difficulties to endorse) in each domain.

Domain	Items	Location	SE	FitResid	ChiSq	Prob
Family interaction	1	0.353	0.088	−1.168	1.65	0.800
	4	0.175	0.102	−1.115	10.23	0.037
	5	0.086	0.082	0.255	3.79	0.435
	2	−0.036	0.086	−0.043	2.53	0.639
	3	−0.146	0.084	−0.082	3.33	0.504
	6	−0.432	0.083	1.848	4.91	0.297
Parenting	9	0.224	0.093	−1.275	4.92	0.296
	7	0.19	0.096	−1.300	6.30	0.178
	8	0.142	0.095	0.806	2.18	0.703
	10	0.062	0.093	−1.426	2.08	0.722
	12	−0.299	0.096	1.328	3.63	0.459
	11	−0.319	0.098	0.819	9.35	0.053
Emotional well-being	13	0.117	0.089	1.468	4.62	0.328
	16	0.028	0.068	1.240	4.56	0.335
	15	0.007	0.068	−1.294	4.63	0.328
	14	−0.153	0.071	−1.288	11.15	0.025
Physical/material well-being	19	0.332	0.102	−1.748	6.23	0.183
	17	0.097	0.096	1.501	4.11	0.392
	20	−0.106	0.095	−2.075	8.08	0.089
	18	−0.126	0.094	0.886	1.39	0.847
	21	−0.196	0.091	1.046	2.98	0.561
Disability-related support	22	0.23	0.079	2.114	3.37	0.498
	23	0.138	0.076	−1.981	7.00	0.136
	24	−0.035	0.075	−1.330	11.68	0.020
	25	−0.333	0.072	0.523	3.17	0.530

SE = standard error.

**Figure 3:**
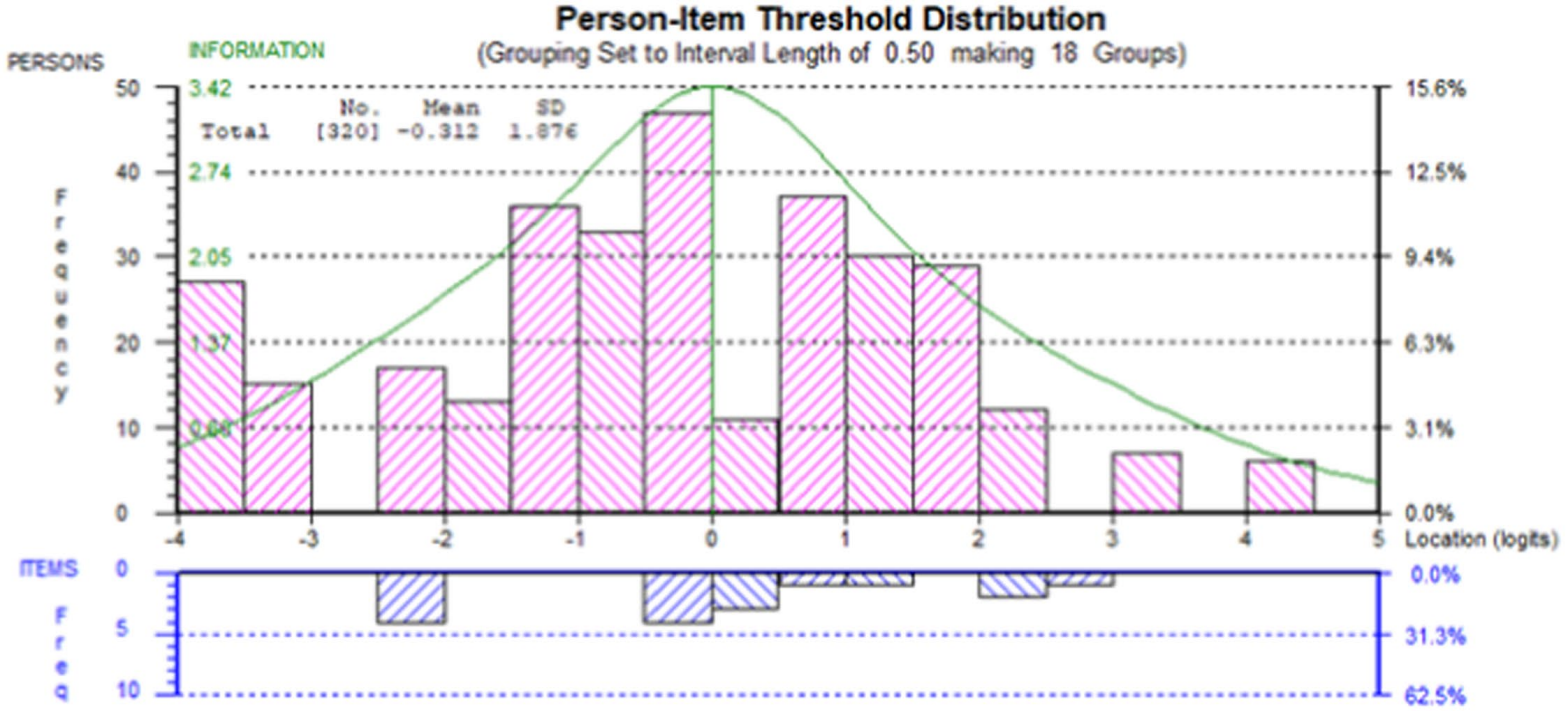
Person-item threshold distribution.

In addition, DIF was checked for all items, and there were no indicators for DIF, regardless of age or sex (Figure 3). Table 1 shows the transformation of raw scores to interval scores. The interval scores ranged from 0 to 100, and the weights of each unit were the same across the scale.

## Discussion

This study aimed to confirm the construct validity of the FQOL-AR using Rasch analysis. The results showed that the 25-item scale was multidimensional. The results support the use of a multidimensional scale. This support the findings of previous studies related the scale factors (Park et al., 2003; Verdugo et al., 2005; Balcells-Balcells et al., 2011, 2020; Isaacs et al., 2012; Meral et al., 2013; Parpa et al., 2016; Chiu et al., 2017; García-Grau et al., 2018; Huang et al., 2018; Waschl et al., 2019; Alnahdi et al., 2021). The results indicated that each of the five subscales had a unidimensional scale. No DIF was detected on any of the scale items, regardless of the gender or age of the participants. In addition, a very good internal consistency of the indicators for the subscales was obtained. These results are partially consistent with studies that concluded the five-factor scale (Hu et al., 2012; Meral et al., 2013; Boehm et al., 2015; Chiu et al., 2017; Alnahdi et al., 2021) and add the dimensionally support at the subscale level, as the first study to provide a support for that. The dimensionality support on the subscale level is an important base that was needed before researchers can calculate mean score (Andrich and Marais, 2019; Alnahdi and Yada, 2020) to represent participant’s level of quality of life on that latent variable (the factor).

The item statistics showed that the best indicator for family interaction was item 1, “*My family enjoys spending time together*.” Item 9, “*Family members teach the children how to get along with others*,” was the best indicator of satisfaction with parenting. Similarly, item 9 had the lowest mean score on this domain in Boehm et al. (2015), which can be seen as an indicator that it was difficult for families to be satisfied in the US sample.

The best indicator of emotional well-being satisfaction was item 13, *“My family has the support we need to relieve stress.”* Item 19, “*My family gets medical care when needed*,” was the best indicator of satisfaction with physical/material well-being. The best indicator for satisfaction with disability-related services was item 22, “*My family member with special needs has [the] support to make progress at school or workplace.*” Knowing the characteristics of the items individually will benefit those interested in taking a small number of items for various reasons by taking the best indicators for each domain.

In addition, this study adds initial support for the use of the mean score of the five items for each of the five subscales. It is important to note that the unidimensionality of the 25 items BCFQOL is not supported at the item level. This study supported the unidimensionality of each of the five subscales, which is in line with Hoffman et al.’s (Hoffman et al., 2006) statement regarding the original version of the scale that “each of the five subscales was shown to be unidimensional and internally consistent” (p. 1080). The study findings support the multidimensionality of the BCFQOL-AR, which is consistent with the notion that the FQOL is a multidimensional construct, as has been discussed in several studies (Brown and Brown, 2004; Hoffman et al., 2006; Boehm et al., 2015). This is consistent with Cheng et al. (2009) statement that “educational and psychological tests are often composed of multiple short subtests, each measuring a distinct latent trait” (p. 369). In sum, this could imply that these results support using the mean score at the subscale level, and that the total score on each subscale represents the underlying variable for that subscale. Also, these results support the use of any of the subscales of the scale as an independent unit if the researcher needs to collect data related to one of the five aspects of the scale without the need to use other subscales. These results are based on the results of the data from the Arabic version, nonetheless, it is expected that we will obtain comparable results for the other samples, especially that the psychometric properties of the Arabic version of the factorial analysis results (Alnahdi et al., 2021) were consistent with the original version (Hoffman et al., 2006).

### Limitations

There is a limitation that needs to be considered while interpreting this study results. The sample was from one region of the country, therefore, future research with samples from other regions will be important to confirm this study results.

## Conclusion

This study identified indicators that support the multidimensionality of the BCFQOL scale based on the Rasch analysis results as regards the dimensionality test for the scale as a whole (25 items). It supports the multidimensionality of the BCFQOL scale and the notion that each of the five subscales is a unidimensional scale. Therefore, obtaining a total score at the subscale level is recommended. In addition, it showed that each of the five subscales of the BCFQOL can be used alone. For future research, data from different countries that use the BCFQOL would be one way to examine it across different countries. This will also allow us to examine the item scores in different countries. In conclusion, this is the first study that provides statistical evidence using Rash analysis that supports the use of means at the level of subscales of the BCFQOL scale.

## Data availability statement

The raw data supporting the conclusions of this article will be made available by the authors, without undue reservation.

## Ethics statement

The studies involving human participants were reviewed and approved by IRB Committee at Prince Sattam bin Abdulaziz University. The patients/participants provided their written informed consent to participate in this study.

## Author contributions

The author confirms being the sole contributor of this work and has approved it for publication.

## Funding

This work was supported by Deanship of Scientific Research at Prince Sattam Bin Abdulaziz University (RG #2021/02/18510).

## Conflict of interest

The author declares that the research was conducted in the absence of any commercial or financial relationships that could be construed as a potential conflict of interest.

## Publisher’s note

All claims expressed in this article are solely those of the authors and do not necessarily represent those of their affiliated organizations, or those of the publisher, the editors and the reviewers. Any product that may be evaluated in this article, or claim that may be made by its manufacturer, is not guaranteed or endorsed by the publisher.
